# The Antibacterial Activity of Lavender Essential Oil Alone and In Combination with Octenidine Dihydrochloride against MRSA Strains

**DOI:** 10.3390/molecules25010095

**Published:** 2019-12-26

**Authors:** Paweł Kwiatkowski, Łukasz Łopusiewicz, Mateusz Kostek, Emilia Drozłowska, Agata Pruss, Bartosz Wojciuk, Monika Sienkiewicz, Hanna Zielińska-Bliźniewska, Barbara Dołęgowska

**Affiliations:** 1 Department of Diagnostic Immunology, Chair of Microbiology, Immunology and Laboratory Medicine, Pomeranian Medical University in Szczecin, 72 Powstańców Wielkopolskich Avenue, 70-111 Szczecin, Poland; mkosa9406@gmail.com (M.K.); bartosz.wojciuk@pum.edu.pl (B.W.); 2 Center of Bioimmobilisation and Innovative Packaging Materials, Faculty of Food Sciences and Fisheries, West Pomeranian University of Technology Szczecin, Janickiego 35, 71-270 Szczecin, Poland; lukasz.lopusiewicz@zut.edu.pl (Ł.Ł.); emilia_drozlowska@zut.edu.pl (E.D.); 3 Department of Laboratory Medicine, Chair of Microbiology, Immunology and Laboratory Medicine, Pomeranian Medical University in Szczecin, 72 Powstańców Wielkopolskich Avenue, 70-111 Szczecin, Poland; agata.pruss@pum.edu.pl (A.P.); barbara.dolegowska@pum.edu.pl (B.D.); 4 Department of Allergology and Respiratory Rehabilitation, Medical University of Łódź, Żeligowskiego 7/9, 90-752 Łódź, Poland; monika.sienkiewicz@umed.lodz.pl (M.S.); hanna.zielinska-blizniewska@umed.lodz.pl (H.Z.-B.)

**Keywords:** MRSA, lavender essential oil, octenidine dihydrochloride, synergistic activity, FTIR

## Abstract

In the post-antibiotic era the issue of bacterial resistance refers not only to antibiotics themselves but also to common antiseptics like octenidine dihydrochloride (OCT). This appears as an emerging challenge in terms of preventing staphylococcal infections, which are both potentially severe and easy to transfer horizontally. Essential oils have shown synergisms both with antibiotics and antiseptics. Therefore the aim of this study was to investigate the impact of lavender essential oil (LEO) on OCT efficiency towards methicillin-resistant *S. aureus* strains (MRSA). The LEO analyzed in this study increased the OCT’s susceptibility against MRSA strains. Subsequent FTIR analysis revealed cellular wall modifications in MRSA strain cultured in media supplemented with OCT or LEO/OCT. In conclusion, LEO appears to be a promising candidate for an efficient enhancer of conventional antiseptics.

## 1. Introduction

*Staphylococcus aureus* is a commensal bacterium that can colonize the skin and the mucoses of humans, but is also a pathogenic microorganism responsible for many types of infections. The pathogenicity of this bacterium is primarily associated with the variety of virulence factors like enzymes and toxins [1]. Virulence factors combined with the efficient evasion mechanisms make *S. aureus* a formidable opponent. Asymptomatic carriage, mostly present in the nasal vestibule, may directly influence the development of infection under favorable circumstances. Skin and soft tissue infections (SSTIs) are the most common forms of *S. aureus* etiology [2]. These occur in both outpatients and inpatients. These infections are associated with the disruption of natural protective barriers in the skin and mucous membranes. After invasion, the bacteria multiply, the expression of their virulence genes as well as toxins production increases, and this results in the development of clinical symptoms such as SSTIs and surgical site infections (SSIs) [3].

In each of these, microbes can enter the vascular bed and cause severe systemic infection. This applies especially to inpatients in intensive care units and surgical departments but also to hemodialyzed outpatients for whom long lasting endovascular catheterization is applied. For these patients, experiencing circulatory failure, respiratory failure, severe surgical trauma, hypothermia, or hypovolemia increases the degree of tissue hypoxia. Additionally, diabetes mellitus frequently coexisting with hemodialysis is predisposed to bacterial colonization in the sites exposed to iatrogenic skin damage. As a result, the risk of systemic infection increases significantly [4].

As *S. aureus* potentially contributes to the skin microflora, the horizontal transmission of pathogenic strains appears to be critical. Most of the aforementioned infections can spread easily via skin-to-skin contact or via contaminated everyday use items. Consequently, it is crucial to deliver efficient and safe hygiene measures in order to disrupt the transmission process.

Moreover, due to increasingly common resistance to β-lactam antibiotics among *S. aureus*, the treatment of infections with these microorganisms, including the SSIs, has become more challenging. Methicillin-resistant *Staphylococcus aureus* (MRSA) strains are not only found in hospital environments or in inpatients, but can also develop in outpatients [5]. In inpatients with a high risk of colonization by MRSA due to having implanted artificial valves or vascular grafts, vancomycin is given as an alternative drug [6]. In order to avoid complications, various types of antiseptic agents are also used in wound care. One of them is *N,N***′**-(1,10-decanediyldi-1[*4H*]-pyridinyl-4-ylidene)-bis-(1-octanamine) dihydrochloride, also known as octenidine dihydrochloride (OCT) [7]. OCT is a cationic active compound that exhibits a broad bactericidal spectrum, including MRSA. This antiseptic agent works by interacting with bacterial cell structures, which consequently results in lysis and cell death. OCT is light-resistant, and is chemically stable in a broad range of pH (1.6–12.2) and temperatures [8]. In addition to high antibacterial efficacy, OCT neither adversely affects epithelial cells, nor impedes the wound healing process. OCT is used only topically and is not absorbed into general circulation, so it does not cause any systemic effects. Due to its properties, OCT works well when applied to wounds, mucous membranes, and skin. OCT shows a synergistic effect with phenoxyethanol, hence phenoxyethanol as an aqueous solution in combination with OCT is applied in medical practice [7,9], e.g., to decolonize vulnerable patients with MRSA, which is an indispensable element of hospital-acquired infection prevention.

Still, the problem of bacterial resistance also applies to antiseptics such as OCT. Hardy et al. [10] observed a correlation between the use of these antiseptics and a staphylococcal sensitivity decrease. They stated that Minimum Inhibitory Concentration (MIC) and Minimum Bactericidal Concentration (MBC) values of OCT increased rapidly after OCT’s introduction to widespread use. The authors pointed out the mutations in *norA* and *norB* genes encoding efflux pump proteins as a possible reason for bacterial tolerance towards antiseptics. Hence, investigating the preparation methods which support the activity of antiseptics seems to be an important and interesting research area.

This is particularly so with regard to common exposure and the severity of staphylococcal infections as described above. Essential oils (EOs) represent a major example of this [11]. Firstly, the combination of EOs and antiseptic agents can contribute to reducing the risk of infection in healing wounds caused by MRSA strains. Secondly, a synergistic effect between the active compounds can enable a dose reduction and a concomitant alleviation of side effects typically associated with these EOs and antiseptic agents. Finally, some EOs have a pleasant fragrance which can provide psychological benefits facilitating wound healing.

It seems that lavender essential oil (LEO) extracted from the flowering tops of *Lavandula angustifolia* Mill. (*Lamiaceae*) is a promising candidate for a natural product which can increase the synergistic effect of some antiseptic agents such as OCT. LEO has a wide range of applications in pharmaceutical products and as a fragrance ingredient in the cosmetics industry [12]. It has been also proven that LEO has beneficial immunomodulatory effects on wound healing [13]. In addition, this oil has various pharmacological effects described in the available literature, such as antibacterial, antifungal, antioxidant, anxiolytic, anticonvulsant, and anticholinesterase properties [14,15,16,17,18,19]. According to Malcolm and Tallian [20], LEO is classified as Generally Recognized as Safe (GRAS) by the Food and Drugs Administration (FDA) (21CFR182.20 2015).

The exact mode of action of LEO is still not fully recognized. It is hypothesized that it influences bacterial wall ultrastructure and therefore modifies whole bacterial cell susceptibility. Hence, the aim of this study was to investigate the influence of LEO on the antibacterial activity of OCT against MRSA strains. Special attention was paid to the possible effect of LEO on bacterial cell wall modification.

## 2. Results

### 2.1. Chemical Analysis of LEO

**Figure 1:**
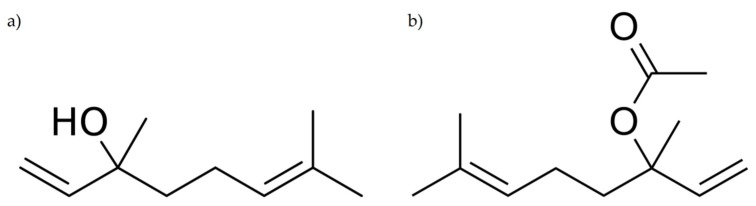
The chemical structures of the main compounds of lavender essential oil: linalool (**a**) and linalyl acetate (**b**).

The qualitative and quantitative chemical composition of LEO analyzed using GC-FID-MS are listed in Table 1. The total number of compounds identified in LEO was 29, representing 98.5% of the total oil content. The remaining compounds (1.5%) appeared in trace amounts. The main constituents of tested LEO were linalool (34.1%) and linalyl acetate (33.3%) (Figure 1) followed by lavandulyl acetate (3.2%), (*Z*)-β-ocimene (3.2%), (*E*)-β-ocimene (2.7%), β-caryophyllene (2.7%), 1,8-cineole (2.5%), terpinene-4-ol (2.5%), and myrcene (2.4%).

### 2.2. The Antibacterial Activity of Chemicals against MRSA Strains

As determined using the microdilution method, the control strain was susceptible to both LEO and OCT. The obtained MIC values were 1.95 ± 0.00 µg/mL and 18.29 ± 7.92 mg/mL for OCT and LEO, respectively. It was also found that both OCT and LEO showed antibacterial activity against MRSA clinical strains. The MIC of OCT inhibiting growth of these strains ranged between 3.52 ± 0.00 µg/mL to 3.91 ± 0.00 µg/mL, whereas the MIC of LEO was slightly higher (13.72 ± 0.00 mg/mL). Moreover, it was also observed that the addition of Tween 80 (1%, *v*/*v*) or DMSO (2%, *v*/*v*) had no impact on the growth of any of the strains. The results of the MICs and MBCs of OCT and LEO against MRSA strains are summarized in Table 2.

### 2.3. Synergistic Effect of LEO and OCT

The study showed that LEO presented synergistic activity in combination with OCT against MRSA reference strain and clinical isolates (the FICI values ranged from 0.11 to 0.26). The detailed results of a checkerboard assay against MRSA strains are summarized in Table 2.

### 2.4. Effect of LEO Alone and In Combination With OCT against MRSA Reference Strain

#### 2.4.1. Time-Killing Curves

**Figure 2:**
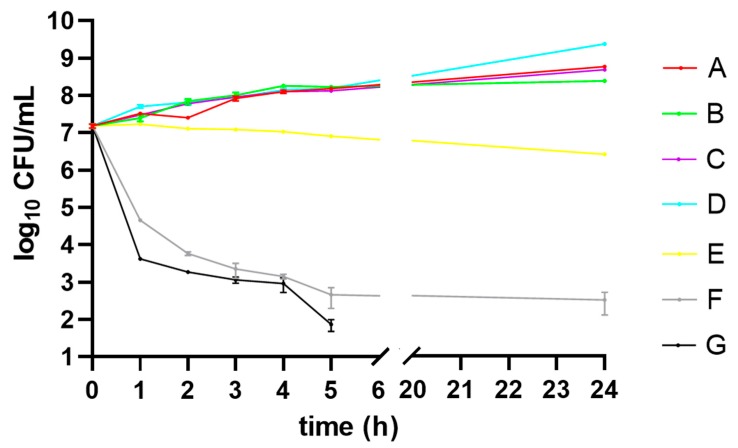
Time-kill kinetics of *Staphylococcus aureus* ATCC 43300 (MRSA) strain grown in Mueller-Hinton broth containing: no chemicals (control—medium A), Tween 80 (medium B), DMSO (medium C), Tween 80 and DMSO (medium D), lavender essential oil (LEO) at subinhibitory concentration (MIC_50_) (medium E), octenidine dihydrochloride (OCT) at subinhibitory concentration (MIC_50_) (medium F), LEO/OCT at subinhibitory concentrations (MICc_50_) (medium G). CFU—colony forming unit.

The time-kill kinetics profile of MRSA reference strain grown in different media (A–G) are shown in Figure 2. The MRSA strain cultured in medium G showed a reduction in the number of viable cells within the first 5 h when compared to the medium E as well as medium F.

#### 2.4.2. FTIR Analysis

**Figure 3:**
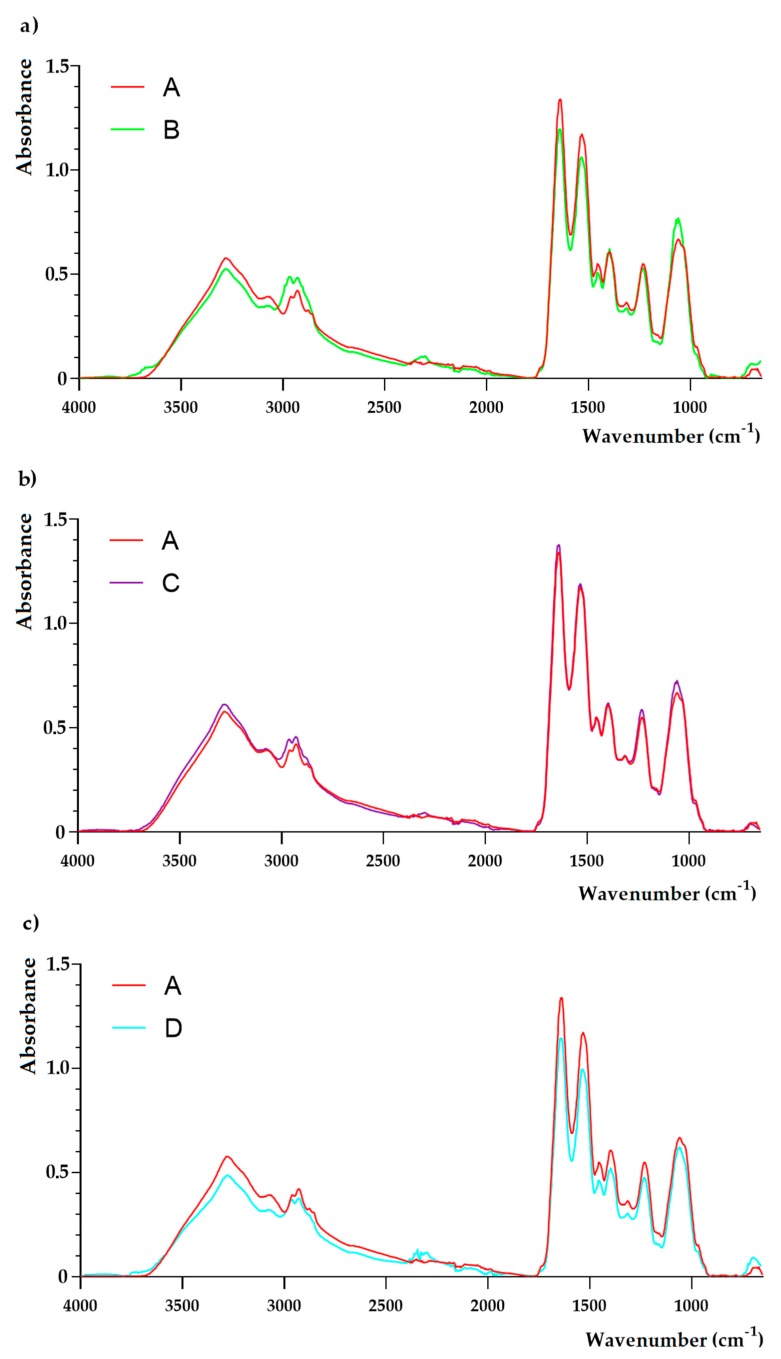

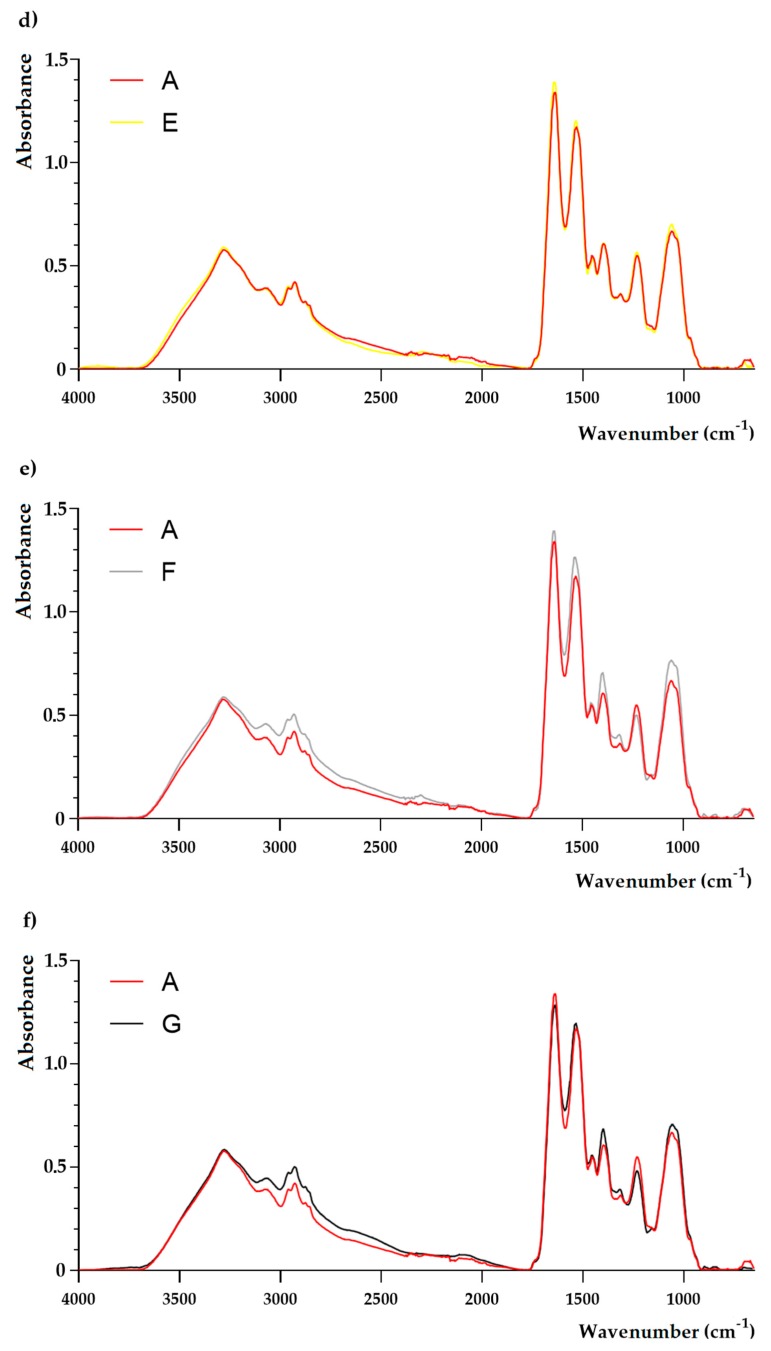
FTIR spectra of *Staphylococcus aureus* ATCC 43300 (MRSA) strain grown in Mueller-Hinton broth containing: no chemicals (control—medium A), Tween 80 (medium B), DMSO (medium C), Tween 80 and DMSO (medium D), lavender essential oil (LEO) at subinhibitory concentration (MIC_50_) (medium E), octenidine dihydrochloride (OCT) at subinhibitory concentration (MIC_50_) (medium F), LEO/OCT at subinhibitory concentrations (MICc_50_) (medium G).

**Figure 4:**
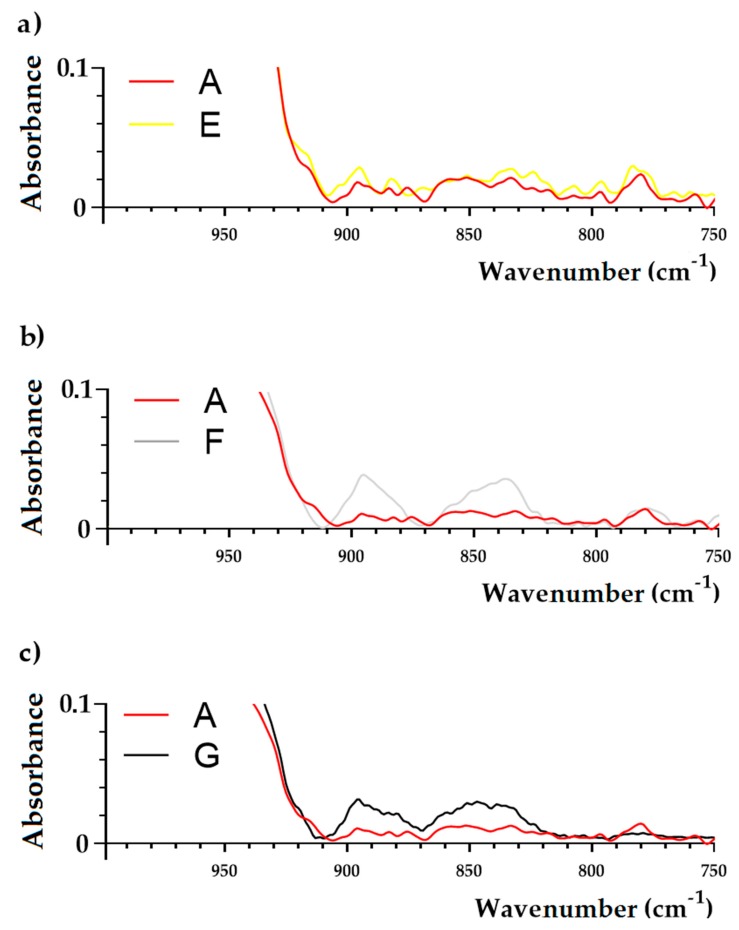
FTIR spectra (in the range: 950–700 cm^−1^) of *Staphylococcus* *aureus* ATCC 43300 (MRSA) strain grown in Mueller-Hinton broth containing: no chemicals (control—medium A), lavender essential oil (LEO) at subinhibitory concentration (MIC_50_) (medium E), octenidine dihydrochloride (OCT) at subinhibitory concentration (MIC_50_) (medium F), LEO/OCT at subinhibitory concentrations (MICc_50_) (medium G).

The complete FTIR spectra of the samples are shown in Figure 3 and Figure 4. No qualitative differences were observed between samples isolated from media B-E and the control sample (medium A). However, the analysis targeting in particular cellular wall components was revealed. The differences in FTIR spectra between the sample isolated from media E-G in comparison to the control sample (medium A) were observed. In the E sample, no changes at 3280 cm^−1^, 2959 cm^−1^, 2927 cm^−1^, 1454 cm^−1^, and 1394 cm^−1^ were noticed. A noticeable growth of absorbance at bands 1636 cm^−1^, 1532 cm^−1^, and 1230 cm^−1^ was observed. Moreover, an increase of absorbance at 1057 cm^−1^ was also observed. Sample F showed a more multi-faceted influence on the chemical composition of *S. aureus* cells (Figure 3). Noticeable growth of absorption peaks were observed. Moreover, the new peaks at 895 cm^−1^ and 837 cm^−1^ were noticed (Figure 4). The FTIR spectrum of a sample exposed to both E and F samples showed that all changes observed in the cells under the influence of both compounds are also separately found when these compounds are used together.

## 3. Discussion

There has been a dramatic increase in bacterial resistance to antibiotics and chemotherapeutics, which limits their therapeutic use. It was also observed that the effectiveness of new antibiotics and chemotherapeutics is rapidly decreasing. Scientific data led to the announcement in 2014 by the World Health Organization of the beginning of a post-antibiotics era. In this study, the activity of commercial LEO from the flowering herb of *L. angustifolia* Mill. (*Lamiaceae*) in combination with OCT against MRSA strains was analyzed. It has been proven that chemical analysis of LEO used in this study met the requirements outlined in the ISO Standard 11024 [21,22].

This study showed that a combination of LEO and OCT increases the effectiveness of this commonly used antiseptic agent against MRSA strains. The combination of OCT and antibiotics such as mupirocin is commonly used to eradicate the nasal carriage of *S. aureus* (especially MRSA) straight before surgical operations, especially cardiac surgery [23]. However, there are interesting data about the interactions of antiseptics with antibiotics. Hübner et al. [7] described the synergistic interaction of OCT incorporated into Mueller-Hinton agar with imipenem (against *Enterococcus faecalis*, *Enterococcus faecium* and *Pseudomonas aeruginosa*) and piperacillin + tazobactam (against *E. faecalis* and *E. faecium*). The same authors reported synergism between chlorhexidine digluconate (CHD) incorporated into Mueller-Hinton agar and piperacillin + tazobactam against *E. faecalis*. However, there are also reports about interaction of widely used antiseptics with essential oils and their main compounds. According to Şimşek and Duman [24], combinations of 1,8-cineole with CHD showed synergistic interactions against the following reference strains: *S. aureus* (ATCC 25923), *Escherichia coli* (ATCC 25922), *E. faecalis* (ATCC 51299), *Klebsiella pneumoniae* (ATCC 700603), and *Candida albicans* (ATCC 90028) (fractional inhibitory concentration indices—FICI values = 0.13–0.38), as well as MRSA clinical isolate (FICI = 0.05). The authors suggest that this combination may be beneficial in skin antisepsis by causing the elimination of microcolonies which are likely to exhibit increased resistance to CHD. Karpanen et al. [25] presented a similar conclusion in that essential oils, in particular eucalyptus essential oil which is more than 90% 1,8-cineole, can be used for an improved skin antisepsis when combined with CHD. They showed that in biofilm, CHD combined with eucalyptus oil demonstrated synergistic activity against the clinical isolate of *Staphylococcus epidermidis*, with an FICI value of 0.19. According to Alabdullatif et al. [26] linalool significantly enhances anti-biofilm activity of CHD with isopropyl alcohol and can potentially be used to improve skin disinfection.

According to literature data, the antimicrobial activity of essential oils depends on the content of terpenoides. Among them, phenolic compounds such as thymol or carvacrol can be distinguished by their strong action. In contrast, terpene alcohols (e.g., geraniol, citronellol, and linalool) and esters (e.g., linalyl acetate) show slightly weaker antimicrobial activity [27]. LEO owes its activity mainly to linalool and linalyl acetate, but it is known that compounds present in lower amounts are important in creating a unique mixture with a particular synergy. In this investigation, it has been proven that combination of LEO containing mostly linalool (34.1%) and linalyl acetate (33.3%) showed a synergistic effect in combination with OCT against methicillin-resistant staphylococci both the reference strain *S. aureus* ATCC 43300 and clinical isolates with FICI values between 0.11–0.26. LEO as a safe (GRAS) natural product of plants could be a good candidate to investigate its application in skin antiseptic formulation. According to literature data, LEO is well-tolerated on the surface of skin and is often administered orally or applied topically in an undiluted form [20]. However, Prashar et al. [28] showed cytotoxic activity of LEO containing mainly linalyl acetate (51%) and linalool (35%) on human skin cells (HMEC-1, HNDF, and 153BR) at a concentration of 0.25% (*v*/*v*). Nevertheless, in this study the most effective combination of LEO and OCT decreased the MIC of LEO from 14.86 ± 3.96 mg/mL (1.49 ± 0.4%) to 1.29 ± 0.49 mg/mL (0.13 ± 0.05%). In our previous study, it was also observed that LEO derived from the same production batch exhibited low cytotoxic activity towards HMEC-1 and glioblastoma cell (T98G) lines [29]. IC_50_ values of LEO against HMEC-1 and T98G lines were 5.15 μL/mL (4.5 mg/mL) and 2.27 μL/mL (1.99 mg/mL), respectively. Moreover, a more efficient killing effect caused by synergistic LEO-OCT pairs at subinhibitory concentrations (MICc_50_) was noticed. It has been shown that after five hours of incubation, there was a noticeable reduction of viable cells when compared to the control medium (without compounds). It was also observed that the addition of Tween 80 (1%, *v*/*v*) and DMSO (2%, *v*/*v*) had no impact on MRSA strains growth inhibition, and this has been also noted previously by Honório et al. [30] and Ferguson et al. [31]. Moreover, Tween 80 at the concentration of 1% is widely used as an emulsifier in cosmetics, pharmaceuticals, and food products, and has been approved by the US Food and Drug Administration for use in selected foods [32].

The present study showed the qualitative differences in FTIR spectra of samples F (Mueller-Hinton broth (MHB) containing OCT at subinhibitory concentration—MIC_50_) and G (MHB containing LEO/OCT at subinhibitory concentration—MICc_50_) in comparison to the control sample (MHB without chemicals). As a result of cultivation of the MRSA strain in MHB containing OCT at a subinhibitory concentration (MIC_50_), two new peaks were observed at 895 cm^−1^ and 837 cm^−1^, which were also noticed in MRSA cells grown in medium F (MHB containing LEO/OCT at subinhibitory concentrations—MICc_50_). Those changes are assignable to C-O-C glycosidic linkages and C-O-P symmetric stretching vibrations in cell wall oligosaccharides and polysaccharides, which may affect the electrostatic interactions with antibacterial molecules [33,34]. In previous studies, lower penetration of anionic antibiotic mupirocin was observed in a mupirocin-resistant MRSA strain [33]. On the other hand, OCT is a cationic, surface active antimicrobial compound (its molecular weight is approximately 624 Da), whose mode of action is based on integration with enzymatic systems. As a result, polysaccharides in the cell wall of microorganisms induce leakages in the cytoplasmic membrane and lead to cell death [7,8,35]. It has two non-interacting cation-active centers in its molecule, which are separated by a long aliphatic hydrocarbon chain [8,35]. Like other cationic antiseptics, OCT’s main target appears to be glycerol phosphates in the bacterial cell membrane. It therefore binds readily onto negatively charged surfaces, such as microbial cell envelopes and eukaryotic cell membranes [7]. Thus, based on FTIR results, it can be assumed that cultivation of MRSA in medium containing subinhibitory concentration (MIC_50_) of OCT resulted in changes in cell wall oligosaccharides and polysaccharides that resulted in the change of electrostatic potential of the cell surface, which therefore may affect OCT antibacterial efficacy. Similarly, the antibacterial activity of essential oils is mainly based on acting on the cytoplasmic membrane, which results in a loss of membrane stability and increased permeability [36]. In general, Gram-positive bacteria are more susceptible to essential oils in comparison to Gram-negative bacteria [36,37]. This can be linked to the fact that Gram-negative bacteria have an outer membrane which is rigid, rich in lipopolysaccharide (LPS), and more complex, thereby limiting the diffusion of hydrophobic compounds through it. This extra complex membrane is absent in Gram-positive bacteria, which instead are surrounded by a thick peptidoglycan wall that is not dense enough to resist small antimicrobial molecules, thus facilitating the access to the cell membrane [36,37,38]. Moreover, Gram-positive bacteria may ease the infiltration of hydrophobic compounds of EOs due to the lipophilic ends of lipoteichoic acid present in cell membrane [36,38]. Since OCT is hydrophobic compound, it requires organic solvent such a as phenoxyetanol in order to be effectively administered [8]. LEO is primarily composed of monoterpenoids and sesquiterpenoids where linalool and linalyl acetate are the most dominant, representing hydrophobic character [39,40]. Studies on the effects of the major chemical constituents of *L. angustifolia*, comprising essential oil, linalool, linalyl acetate, and terpinen-4-ol, indicate that the mechanism of action of these components damages the lipid layer of the cell membrane, which results in bacterial cell leakage [11]. Thus, based on the results of time-killing curve it may be assumed that LEO has a synergistic effect on OCT, thereby enhancing its permeation into bacterial cells.

## 4. Materials and Methods

### 4.1. Bacterial Strains and Growth Condition

The study included three methicillin-resistant *S. aureus* isolates belonging to the collection of the Chair of Microbiology, Immunology and Laboratory Medicine in Pomeranian Medical University in Szczecin, Poland. The strains were isolated from surgical wound infections. The specimens were cultivated on Columbia agar with 5% sheep blood (bioMérieux, Warsaw, Poland), incubated 18 h at 37 °C in aerobic atmosphere, and identified using the biochemical test GP Vitek 2 Compact (bioMérieux, Warsaw, Poland). A *S. aureus* ATCC 43300 (MRSA) strain was used as the control strain in this study.

### 4.2. Chemicals

#### 4.2.1. Chemical Characterization of LEO

Commercial LEO from the flowering herb of *L. angustifolia* Mill. (*Lamiaceae*) was purchased from Pollena-Aroma (Nowy Dwór Mazowiecki, Poland). The LEO was analyzed by gas chromatography-flame ionization detector-mass spectrometer (GC-FID-MS) at the Institute of General Food Chemistry, Łódź University of Technology, Poland using a Trace GC Ultra apparatus (Thermo Fisher Scientific, Waltham, MA, USA) MS DSQ II detectors, and an FID-MS splitter (SGE, Trajan Scientific Europe, Milton Keynes, UK). Identification of compounds in LEO was based on the comparison of their MS spectra with the MS spectra of computer libraries (MassFinder 3.1, Wiley Registry of Mass Spectral Data, and NIST 98.1 [41,42,43] along with the retention indices on a non-polar column (Rtx-1, MassFinder 3.1, Restek Corporation, Bellefonte, PA, USA) associated with a series of *n*-alkanes with linear interpolation (C-9 to C-26).

Concentrations of LEO from 500 to 0.12 µL/mL were prepared by dissolving essential oil in Tween 80 (Sigma-Aldrich, Darmstadt, Germany) (1%, *v*/*v*) and diluting by Mueller-Hinton broth (MHB, Sigma-Aldrich, Darmstadt, Germany).

#### 4.2.2. Octenidine Dihydrochloride (OCT)

OCT with a purity of no less than 98.0% was obtained from Schülke & Mayr GmbH (Norderstedt, Germany). Concentrations of OCT from 500 to 0.12 µg/mL were prepared by dissolving the chemical in dimethyl sulfoxide (DMSO, Loba Chemie, Mumbai, India) (2%, *v*/*v*) and diluting it using MHB.

### 4.3. Determination of Minimum Inhibitory Concentration (MIC) and Minimum Bactericidal Concentration (MBC) of Chemicals

The MIC of LEO or OCT was determined by the broth microdilution method according to the Clinical and Laboratory Standards Institute with a slight modification as described previously [44]. To exclude an inhibitory effect of both Tween 80 and DMSO, the control assays with MHB and MHB supplemented with Tween 80 (1%, *v*/*v*) or DMSO (2%, *v*/*v*) were performed. All tests were carried out in duplicate. At this stage, MIC_50_ of each chemicals against *S. aureus* ATCC 43300 was calculated.

The MBC of chemicals was determined by transferring 20 µL of cultures in higher-than-MIC concentrations on a 96-well microplate contained MHB (100 µL) in each well, and incubating them for 18 h at 37 °C. After this period, the MBC was observed and the concentration on which transparent and verifiable medium could be found was identified. Using the known density of LEO, the final result was expressed in mg/mL.

### 4.4. Checkerboard Method

Combinations of LEO and OCT against MRSA strains were tested by using a previously described checkerboard method [44]. Using the known density of the LEO, the final result was expressed in mg/mL. All tests were performed in duplicate. Within each chemical, the lowest inhibitory concentration was considered as a minimum inhibitory concentration in combination (MICc). At this stage, MICc_50_ of OCT/LEO combination against *S. aureus* ATCC 43300 was calculated. For each replicate, fractional inhibitory concentration indices (FICI) were estimated using the Equations (1) and (2):

(1) $$\mathrm{FIC}\;=\;\frac{\mathrm{MIC}\;\mathrm{of}\;\mathrm{LEO}\;\mathrm{or}\;\mathrm{OCT}\;\mathrm{in}\;\mathrm{combination}\;}{\mathrm{MIC}\;\mathrm{of}\;\mathrm{LEO}\;\mathrm{or}\;\mathrm{OCT}\;\mathrm{alone}}$$

FICI = FIC of LEO + FIC of OCT.(2)

Results were interpreted as follows: synergy (FICI < 0.5), addition (0.5 ≤ FICI ≤ 1.0), indifference (1.1 < FICI ≤ 4.0), or antagonism (FICI > 4.0).

### 4.5. The Influence of LEO Alone and In Combination With OCT on the Chemical Composition of the S. aureus ATCC 43300 (MRSA) Strain

#### 4.5.1. Culture Media Preparation

One colony of the *S. aureus* ATCC 43300 (MRSA) strain was harvested from the pure culture (from Columbia agar with 5% sheep blood), inoculated into MHB, and incubated at 37 °C for 18 h with shaking (200 rpm) and the turbidity adjusted to McFarland standard number 2. Then, a 1 mL MRSA strain suspension was added to 50-mL falcon tubes and filled up with 20 mL of MHB containing: without chemicals (control—medium A), Tween 80 (1%, *v*/*v*) (medium B), DMSO (2%, *v*/*v*) (medium C), Tween 80 (1%, *v*/*v*) and DMSO (2%, *v*/*v*) (medium D), LEO at a subinhibitory concentration (MIC_50_) (medium E), OCT at a subinhibitory concentration (MIC_50_) (medium F), and LEO/OCT at subinhibitory concentrations (MICc_50_) (medium G). The falcons were undergoing an 18 h incubation at 37 °C with shaking (200 rpm). Determination of subinhibitory concentrations (MIC_50_ and MICc_50_) of both LEO and OCT, as well as LEO/OCT combination were calculated in proportion to the MIC_100_ and MICc_100_ values obtained in Section 4.3 and Section 4.4, respectively.

#### 4.5.2. Time-Kill Curve Assay

A time dependent killing assay was performed to determine the killing kinetics based on the study conducted by Kang et al. [45] with a slight modification. The media A–F (25 mL) were inoculated with MRSA to obtain bacterial cells concentrations of 0.5 on the McFarland scale. After inoculation, the test tubes were incubated at 37 °C under shaking conditions (100 rpm). The viable cells were determined by counting the colonies formed from a 100-μL samples that were removed from the cultures at 0, 1, 2, 3, 4, 5, 6 12, and 24 h, which were then serially diluted, spread on Mueller-Hinton plates, and incubated for 24 h at 37 °C. Time-kill curves were constructed by plotting the mean colony counts (Log_10_ CFU/mL) versus the time.

#### 4.5.3. A Determination of Functional Groups in Staphylococcal Cells by the Use of Fourier Transform Infrared (FTIR) Spectroscopy

In order to confirm the presence of particular chemical moieties in MRSA reference strain incubated into different microbiological media (A–G), FTIR spectroscopy analyses was performed as described earlier [34]. FTIR is defined as a method that is sensitive to bond polarization (changes in the dipole moment), which therefore gives strong signals for polar functional groups [46,47].

The obtained spectra were normalized, baseline corrected, and analyzed using SPECTRUM software (v10, Perkin Elmer, Waltham, MA, USA).

## 5. Conclusions

LEO appears to be an efficient enhancer of the well-known antiseptic OCT against MRSA strains. It potentially influences bacterial permeation by modifying the cell wall structure. This is mirrored in phenotypic analyzes of MRSA susceptibility to OCT. Therefore LEO appears to offer therapeutic as well as preventive potential in the post-antibiotic era.

## Figures and Tables

**Table 1:** Chemical composition of volatile constituents of commercial lavender essential oil from the flowering herb of *Lavandula angustifolia* Mill. (*Lamiaceae*).

Compound	RI	Relative Concentration (%)
**Monoterpenes**		
α-Pinene	936	0.1
Camphene	950	0.1
Myrcene	987	2.4
*p*-Cymene	1015	0.2
1,8-Cineole	1024	2.5
Limonene	1025	0.6
(*Z*)-β-Ocimene	1029	3.2
(*E*)-β-Ocimene	1041	2.7
γ-Terpinene	1051	0.1
Terpinolene	1082	0.2
**Monoterpene isoprenoids**		
Linalool	1086	34.1
Camphor	1123	1.2
Izoborneol	1142	0.2
Borneol	1150	1.4
Lavandulol	1151	1.1
Terpinene-4-ol	1164	2.5
*cis*-Dihydrocarvone	1172	0.2
α-Terpineol	1176	1.8
Linalyl acetate	1239	33.3
Lavandulyl acetate	1275	3.2
Neryl acetate	1342	0.8
Geranyl acetate	1362	1.3
**Sesquiterpenes**		
β-Caryophyllene	1421	2.7
Aromadendrene	1443	0.1
(*E*)-β-Farnezene	1446	0.4
Bicyclosesquiphellandrene	1487	0.1
**Sesquiterpene isoprenoids**		
Caryophyllene oxide	1578	0.1
**Esters**		
Oct-1-en-3-yl acetate	1093	0.6
**Ketones**		
Octan-3-one	969	1.3
Total		98.5

RI: Retention index measured relative to *n*-alkanes (C-9 to C-26) on a non-polar Rtx-1 column.

**Table 2:** Fractional inhibitory concentration (FIC) and FIC indices (FICI) of octenidine dihydrochloride (OCT)—lavender essential oil (LEO) pairs against methicillin-resistant *Staphylococcus aureus* (MRSA) strains.

	Bacteria	OCT-LEO	MICo	MBC	MICc	FIC	FICI	Type of Interaction
reference strain	ATCC 43300	OCT (µg/mL)	1.95 ± 0.00	5.21 ± 2.26	0.12 ± 0.00	0.06	0.11	synergy
		LEO (mg/mL)	18.29 ± 7.92	439.00 ± 0.00	0.86 ± 0.00	0.05		
isolates	1	OCT (µg/mL)	3.91 ± 0.00	11.72 ± 5.52	0.12 ± 0.00	0.03	0.16	synergy
		LEO (mg/mL)	13.72 ± 0.00	27.44 ± 0.00	1.71 ± 0.00	0.13		
	2	OCT (µg/mL)	3.52 ± 0.00	7.04 ± 0.00	0.24 ± 0.00	0.13	0.26	synergy
		LEO (mg/mL)	13.72 ± 0.00	27.44 ± 0.00	1.71 ± 0.00	0.13		
	3	OCT (µg/mL)	3.52 ± 0.00	7.04 ± 0.00	0.12 ± 0.00	0.06	0.12	synergy
		LEO (mg/mL)	13.72 ± 0.00	27.44 ± 0.00	0.86 ± 0.00	0.06		

Values are expressed as mean ± standard deviation. MICo, minimum inhibitory concentration of OCT or LEO; MBC, minimum bactericidal concentration; MICc, minimum inhibitory concentration of OCT/LEO combination. FIC index = FIC of OCT + FIC of LEO. FICI < 0.5, synergy; 0.5 ≤ FICI ≤ 1.0, addition; 1.1 < FICI ≤ 4.0, indifference; FICI > 4.0, antagonism. Using the known density of LEO, the final result was expressed in mg/mL.
